# ‘Unfocused groups’: lessons learnt amid remote focus groups in the Philippines

**DOI:** 10.1136/fmch-2021-001098

**Published:** 2021-08-04

**Authors:** Mila F Aligato, Vivienne Endoma, Jonas Wachinger, Jhoys Landicho-Guevarra, Thea Andrea Bravo, Jerric Rhazel Guevarra, Jeniffer Landicho, Shannon A McMahon, Mark Donald C Reñosa

**Affiliations:** 1 Department of Epidemiology and Biostatistics, Research Institute for Tropical Medicine, Muntinlupa City, Philippines; 2 Heidelberg Institute of Global Health, Heidelberg University, Heidelberg, Germany; 3 International Health, Johns Hopkins University Bloomberg School of Public Health, Baltimore, Maryland, USA

**Keywords:** COVID-19, public health, qualitative research, primary health care

## Abstract

The ongoing COVID-19 pandemic has required tremendous shifts in data collection techniques. While an emerging body of research has described experiences conducting remote interviews, less attention has been paid to focus group discussions (FGDs). Herein, we present experiences conducting remote FGDs (n=9) with healthcare workers and caretakers of small children in the Philippines. We used ‘Facebook Messenger Room’ (FBMR), the preferred platform of participants. Despite some success, we generally encountered considerable challenges in terms of recruiting, retaining and moderating remote FGDs, particularly among caretakers of small children. Finding a quiet, private place proved unfeasible for many participants, who were juggling family demands in tight, locked down quarters. Connectivity issues and technological missteps compromised the flow of FGDs and minimised the ability to share and compare opinions. For the research team, remote FGDs resulted in a dramatic role shift for notetakers—from being passive observers to active tech supporters, chatbox referees and co-moderators (when audio disruptions occurred). Finally, we note that remote FGDs via FBMR are associated with ethical complexities, particularly as participants often chose to use their personal Facebook accounts, which can compromise anonymity. We developed and continuously refined strategies to mitigate challenges, but ultimately decided to forgo FGDs. We urge fellow researchers with more successful experiences to guide the field in terms of capturing high-quality data that respond to research questions, while also contending with privacy concerns, both in online spaces, as well as physical privacy despite lockdowns in tight quarters.

**Key points**

- For researchers wanting to undertake remote focus group discussions (FGDs)—particularly among caretakers of young children or in settings with tight living quarters—we caution that identifying quiet spaces with minimal disruptions and strong bandwidth can be hard for participants.
- Filipino participants in our study wanted to use ‘Facebook Messenger Room (FBMR)’ for FGDs, and while we found FBMR to be a promising tool, it also entails concerns related to privacy and anonymity.
- Ensuring 45–60 uninterrupted minutes proved impractical for many participants in our context, and we urge researchers planning to conduct online FGDs to consider their study population’s living arrangements, lockdown degrees, bandwidth bundles, privacy capabilities and choice of platform in order to tailor their research approach.

## Introduction

As qualitative researchers, we rely heavily on human interactions and engagements to reveal a wealth of information pertaining to lived experiences.^1 2^ In light of the emergence of various tools for online data collection and the difficulty of conducting data collection in person during the ongoing COVID-19 pandemic, several scholars have offered guidance on methodological and practical adaptations when conducting remote interviews.^3 4^ Herein, we complement these lessons with insights on another key methodology for qualitative research—focus group discussions (FGDs).

FGDs involve bringing together people with similar experiences or backgrounds to explore a specific topic of interest, encouraging them to talk and compare ideas as a means to capture holistic views and social norms.^5 6^ This form of data collection emphasises (sub)cultural values or group norms,^5^ making the technique a valuable component within the qualitative toolbox.^7–10^

Traditionally, FGDs, like most qualitative research, have relied on in-person interactions. But as technology becomes more accessible, researchers have begun testing and refining remote communication options, including remote FGDs.^11–20^ Several studies have conducted remote FGDs, for example, using platforms such as WhatsApp (among young, digitally fluent individuals in Singapore^16^ and Kenya^21^), Zoom (among surgical patients in the USA^22^), or Facebook (FB) secret groups (among mothers in the USA^13^ and among military spouses in Australia^19^). Some scholars also developed their own platforms to conduct remote FGDs, including chat rooms developed on a web-based platform with young persons in Sweden^18^ and the use of Computer-Mediated Communication methods with transgender women in the USA.^17^ These methods were identified as promising alternatives to in-person FGDs, but mostly built on experiences from populations within high-income countries or populations within low/middle-income countries (LMICs) who are young and digitally savvy. Guidance on different platforms and their potential to reach populations in LMICs with a lower digital literacy is limited.

In this paper, we discuss our experiences conducting remote FGDs using the newly developed platform ‘FB Messenger Room’ (FBMR), and the trade-offs in choosing this platform as opposed to others. We also present our general experiences conducting remote FGDs, including how we ourselves initially struggled with this shift, how the notetaker’s role in the FGDs changed, and the differences in recruiting and retaining participants.

### ‘Project SALUBONG’ and our research team

The FGDs described in this article were conducted as part of a larger mixed-method study on vaccine hesitancy in the Philippines.^23^ Using human-centred design, the research examines how families and providers feel about vaccines, how their attitudes and perceptions of vaccines have changed over time, and how individuals feel about an intervention to promote vaccine confidence.

The members of our research team have previously designed, implemented, and evaluated health interventions to address neglected and infectious diseases (eg, childhood pneumonia, leprosy, malaria, helminths, schistosomiasis, etc) across health facilities in the Philippines drawing on a range of qualitative techniques. The two lead authors of this study (MFA and VE), who led the FGDs presented here, have more than two decades of combined experiences moderating or facilitating in-person FGDs.

### Choice of platform and preliminary considerations

Our FGDs were initially planned to be conducted in person. However, in March 2020, the Philippines was placed under community lockdown, including movement restrictions in Metro Manila and neighbouring regions (including Calabarzon region, which is our study site).^24^ This abrupt shift required Filipinos, especially employees and students, to heavily depend on the internet for remote learning and working.^25^

In this context, and considering the timely nature of our research topic, we decided to shift data collection online. Initially, we considered using Zoom to conduct FGDs as it provides a range of privacy controls and features, and as our team was already familiar with it. However, based on previous experience when conducting remote in-depth interviews (IDIs), we decided to give participants the option to choose among several platforms including: FB messenger, Zoom, Skype and Google Meet.^3^ Our target participants described a high level of familiarity and general preference for FB Messenger, which made it our top choice.

FBMR, introduced by FB in April 2020, allows users to create a room and invite anyone, with or without an FB account, to join a video call. Designed to compete with other platforms,^26 27^ FBMR presents an alternative for hosting group calls with up to 50 people, with no limits in terms of call duration and with built-in controls for privacy and security.^26 27^ With these built-in controls, administrators can lock rooms and remove individual participants. Participants can leave rooms at any time and report rooms to FB administration. However, FBMR lacks end-to-end encryption,^28^ which is offered by similar applications,^29^ and poses a number of privacy concerns outlined below (see the ‘Privacy settings and ethical complexities: a word of caution’ subsection below).

Platforms and services provided by FB are highly accessible and accepted in the Philippines. As of January 2020, the Philippines has an estimated 73 million social media users (among a total population of 107 million as of 2019^30^), an 8.6% increase from April 2019,^31^ with an expected increase to 88 million by 2025.^32^ Filipinos spend an average of 10 hours a day online (more than any other country) with a rising dependence on mobile internet and smartphones.^33 34^ Philippine phone network providers offer packages that compel FB usage (ie, free use of FB, but limited to browsing, posting, liking and sharing FB posts, or steeply discounted data packages that entail unlimited use of FB with certain data premiums).^35–37^ This has resulted in a tremendous spread of FB in the country, which is further accelerated by the efforts of news sources, political actors and social networks to engage directly with people through this platform.^38^ As at least one prominent observer has noted that in the Philippines, the use of the internet is nearly synonymous with the use of FB.^39 40^

We conducted a detailed comparison of the two leading potential platforms, FBMR and Zoom (see online supplemental table 1), comparing relevant aspects such as data usage consumption, subscription requirements and fees (which would determine affordability), as well as privacy settings and additional features such as session recording. Based on these considerations and the fact that most participants within our broader study consistently expressed preference for FB as a platform, FBMR emerged as the preferable tool. At the time of writing, we have conducted nine FGDs using FBMR; four with community health workers (n=20; aged 31–60 years), and five with parents (n=27; aged 16–55 years), who have previously refused or accepted vaccines for their children (divided by decision-making pattern).

10.1136/fmch-2021-001098.supp1 Supplementary data

### Preparing and conducting FGDs on FBMR

Prior to shifting FGDs to FBMR, we undertook the following training and preparation exercises for our team: (a) a brief introduction by an information technology (IT) expert (JRG) on FBMR’s features and how to navigate FBMR including how to set preferences for the session, how to invite participants to join and how to be in control during the session; (b) a pilot run within the team via FBMR; and (c) the IT expert mentoring and assisting with the workarounds during the first FGDs. Our experiences in the broader research project with general participant recruitment and data collection preparation are outlined elsewhere^3^; we therefore focus on setting up and executing FGD sessions (see table 1).

**Table 1:** Challenges and adaptations of remote FGDs via FBMR

Remote FGD procedure	Adaptations and recommendations
**Setting up FGD session**	
Obtain informed consent (individually, online, prior to FGD session). Search and verify FB Messenger accounts of participants. Create FBMR (‘Anyone with the Link’ option should be selected when creating the room). Copy and send meeting link to participants. Send participants a reminder message (ie, short background of the activity, date, time). Confirm participants have accepted the invitation on their FB Messenger prior to the session.	Provide participants with load cards/mobile data credit (ensures connectivity of participants and minimises their expenses). Advise participants to look for a quiet private place, if available. Ask participants for their FB Messenger account name and any description of their profile picture (eg, photo colour, background, wearing hat, etc) to ensure that only study participants are invited to the room. Provide those without an FB Messenger account with the meeting link and information on how to join via text or any other messaging application. Advise participants to update FB Messenger application prior to the FGD to avoid difficulties when trying to join the FGD.
**Executing the FGD**	
Anyone with the link can automatically join, so ensure that only verified participants are accepted. Discuss session rules, highlighting those pertinent to an online format (eg, not recording the session, no photos or screen grabs). Introduce yourself as the moderator and the notetaker (as well as any additional study staff, such as IT specialist).	Make use of the ‘mute participants’ function (available to the person who created the room, that is, the notetaker) to minimise background noise. However, only participants can unmute themselves (remind them to unmute when answering). Be attentive for cues indicating participants being distracted (eg, doing household chores or activities at the side). Call them, ask their viewpoints, and bring them back to the conversation. Use ‘roll calls’ when necessary. Type questions in the chat (notetaker) in case some participants have difficulties hearing the questions.
**End of session**	
Close the room (click ‘end’). Delete any remaining messages in the room group chat.	Keep track of participants leaving the meeting early, and follow-up with them as needed. Note that the in-meeting group chat remains accessible even if the room has ‘ended’ requiring the research team to delete the messages to ensure confidentiality.

FB, Facebook; FBMR, FB Messenger Room; FGDs, focus group discussions; IT, information technology.

### Acquiring consent, recording and storing of data

Participants provided written informed consent individually prior to the FGD; consent forms stated that discussions would take place online. Once participants agreed to participate, they were sent an informed consent form in advance via courier or with the help of local healthcare workers. The process of signing consents was captured during individual recorded FBMR video calls, explaining every part of the consent form and that it would be conducted online using the same platform. The process concluded with a ‘selfie consent’ where the respective participants took a picture while holding their signed consent sheets.^3^

Video and audio recordings were recorded using Movavi Screen Capture (Movavi, V.11), and a physical audio recorder as backup. All data gathered were anonymised, number coded and stored in password-protected computers, and all data will be destroyed after the study completion and publication of findings.

## General considerations regarding remote FGDs

### Notetaker: from ‘passive’ to ‘active’

The role of a notetaker in an in-person FGD is to take detailed notes during the session, and to capture non-verbal cues and information.^41^ Over the course of our remote FGDs, we experienced several circumstances where the notetaker had to assume a more active role.

From setting up the FGD room to following up with participants after a session’s end, the notetaker oversaw logistics: creating the room, inviting participants, video or audio recording the session, contacting the IT on-call in the event of technological problems during sessions and deleting messages in the in-meeting chat box to prevent participants from taking screen grabs of the conversations in the chat. We found that it was often distracting or intrusive to have an IT person in the FGD (as we felt that it created an imbalance in terms of number of research staff vs number of participants and it was challenging in terms of coordinating staff availability), so our notetaker needed to become conversant on IT troubleshooting. The notetaker was in charge of all in-meeting controls (such as muting participants and removing participants) and followed up with participants who lost connection or had technical problems while the session was underway. Additionally, in cases when the moderator had sudden declines in signal strength or stability, the notetaker stepped in to moderate the discussion. Describing this experience, our notetaker used phrases such as ‘exhausting and stressful’, ‘total madness’, ‘a beautiful disaster’ or ‘like herding cats’.

One limitation of FBMR that transpired during FGDs was that only six participants are visible to the moderator on the computer screen at any time. The notetaker therefore had to assist the moderator in informing who among the other participants wanted to contribute to the discussion. At the end of the meeting, notetakers were responsible for closing the room and the safe keeping of session recordings on top of their typical duties as notetakers (noting non-verbal cues, gauging talking time of participants and following the flow of conversation, etc).

### The challenge of recruiting, retaining and engaging participants

Initially, given our preparation efforts, our experiences conducting remote IDIs^3^ and our proposed workarounds for FGDs, we felt hopeful that remote FGDs were feasible. However, challenges began even at participant recruitment. The recruitment of participants, particularly of parents of under-5 children, and FGD scheduling were considerable challenges. We relied heavily on healthcare workers to identify participants from local communities, but we felt that this was a restrictive approach compared with community canvassing and snowball methods we would normally employ. We repeatedly experienced participants backing out last minute (citing reasons such as the need to tend to their children, not having time or noting that all family members are present at the houses due to community lockdowns). In community-based FGDs, finding families to replace ‘no shows’ can be mitigated by recanvassing a neighbourhood and inviting participants, which is challenging in online engagements. Furthermore, we found that in in-person FGDs, participants are less likely to be no shows or to prematurely depart sessions.

In our remote FGDs, caretakers of small children who signed in from home were especially likely to be distracted. Several mothers described an inability to hear with children present, feedback issues and a temptation to use this ‘alone time’ to check social media updates. FGDs with healthcare workers (even those who are parents of small children) were less often disrupted, which we attribute to the fact that these participants could join from their workplace (in health centres). Across participant types, maintaining interest in the subject matter proved challenging, which participants attributed to technical difficulties that made following the flow of FGDs difficult. We tried to send data bundles, troubleshoot with IT, shorten our guide and simplify our questions, but these did not sufficiently address the problem. We found that we could not gather consensus or further probe on issues because those participants who were not speaking often were not following the conversation. This experience of having trouble maintaining interest and engagement throughout the FGD contrasted sharply with our experiences with remote IDIs^3^ where participants could be guided one on one through technical troubles, issues in terms of distraction were much lower, and we generally felt that the data quality was on par with in-person endeavours. In this regard, we felt that many of the measures used to show FGD participants that they are being listened to, and that their opinions and insights matter, especially maintaining eye contact and singular focus on the participants, were very challenging in remote FGDs.

### Privacy settings and ethical complexities: a word of caution

We appreciate substantive and important debates regarding privacy settings of FB (eg, end-to-end encryption of video calls, data protection and security, fake accounts, etc).^42 43^ We included steps to mitigate risks and privacy concerns including: (a) screening of the participants prior to invitation (and continuously checking for uninvited participants to be able to remove them immediately, which proved unnecessary in our FGDs); (b) closing the room and deleting in-meeting chats at the end of the session; and (c) creating and communicating ground rules to all participants before the start of the session, that is, no third-party application screen sharing, screen capture or video recording. We acknowledge the possibility that participants may use third-party recording applications to record FGD sessions, or they may screen grab messages amid in-meeting chats. As a precaution, we did not allow participants to type their answers or ideas in the chat (chat replies were allowed only in instances when sound quality was compromised, and clarification was necessary). In general, these concerns mirror ethical and privacy concerns that we view as applicable to all FGDs,^16–18 21^ where breaches of confidentiality, including unsolicited recording, are possible. Because it is always the responsibility of research teams to take precautions to minimise such risks, we do not view these as fundamental reasons to reject remote FGDs in FBMR.

We are also aware of ethical considerations that relate to identity and identifying features of both FGD participants and the research team. In regard to the former, we recruited participants with the help of community healthcare workers, who established first contact. After confirming (via phone) participants’ desire to partake, and after receiving their permission to contact them via FB Messenger and to confirm their identity via their publicly available FB profile, we reviewed this information to confirm that this appeared to be the same person invited to the data collection activity. This helped us to avoid engagement with fake accounts. Nevertheless, screening participants’ public FB profiles can raise concerns regarding the privacy of these data, and how to systematically limit the consideration of these data to the confirmation of participants’ identity. In terms of our own identity, we decided to use our personal FB Messenger accounts with profile pictures, as we perceived it to be a more authentic and legitimate approach compared with creating an official FB identity for the study. Nevertheless, the existence of an online identity and the manner in which this online persona and presence may affect our engagement with participants and vice versa merit consideration.

In the interest of promoting a sense of ease, we urged participants to consider the following privacy options: (1) create and use a fake account for the FGD (as long as we could verify their accounts prior to the scheduled remote FGD); (2) rename their existing accounts at the start of the session (reverting to their original account name after the close of the session if they wanted); and/or (3) use private browsers in mobile phones to maintain anonymity towards other participants in the session if they do not want to enter the room using their publicly available FB profile.^44^ However, in the FGDs conducted to date, no participants decided to use one of these suggested anonymity-enhancing approaches.

## Discussion

Collecting research data amid the pandemic offers opportunities for qualitative researchers to develop new strategies to engage individuals residing in hard-to-reach settings.^45^ The experiences outlined here, especially the difficulties to keep participants engaged over the course of the FGD, and our perception that the data obtained would not allow comprehensive answers to our research questions, led us to ultimately forgo remote FGDs. In lieu of FGDs, we proceeded with IDIs among community leaders and experts in social media, public relations and social marketing, who we felt could discuss social norms and community-held beliefs in meaningful detail.

Similar to our experiences, researchers in the USA have outlined issues of poor voice and video quality, and unreliable internet access,^20 22^ which are hard to mitigate since these depend on the participant environment and are beyond the control of the researchers. Despite these issues, other studies have also tried to develop the use of online audiovisual technologies or applications as they closely emulate face-to-face interactions.^20 22 46 47^ Studies have highlighted the need to employ practical adaptations such as reminders, prior assessments of participants’ technological capacity and provision of technical support as needed.^20 22 46 47^ In our experience, there were minimal technical difficulties encountered in each session, and this can be attributed to the chosen platform.^22 46^ The provision of technical support from an IT specialist or in our case, the notetaker, proved to be sufficient in handling technical difficulties on both ends. Our pragmatic approach of limiting the number of participants in each focus group mirrors approaches of other research teams.^20 22 46–48^ Limiting the number of participants eases participant management and engagement but we nevertheless could not overcome challenges related to the visibility of participants on the computer screen. Toggling screens to see several participants entails forgoing an ability to watch everyone at once; we therefore sensed that we were missing valuable non-verbal cues, which is vital to successful FGDs.

The technical aspects discussed in this paper were minor contributors to our decision to forego FGDs. Broader environmental aspects proved to be a major deciding factor, one that we as researchers did not fully appreciate until we encountered the aforementioned challenges. The drawbacks of higher dropout rates and participants being distracted by their physical environment have been echoed in studies from the UK^46^ and the USA.^47^ In our experience, participants who were distracted during the session were more prone to dropout early, giving other participants leeway to do the same. Additionally, while background noise could be mitigated by muting participants,^22 46 47^ distractions brought about by video projections (in our case, doing household chores, putting children to sleep and on-screen appearances by others in the household) proved to be challenging and could not easily be mitigated without compromising discussion.^46^ Further evaluation is needed with respect to participant environment, how to better mitigate challenges in advance and whether a forced removal from remote FGD sessions should be imposed, in the event that uncontrollable distractions arise.^46^

While some researchers have argued that remote FGDs could be a promising alternative or complement to in-person FGDs,^20 22 47^ our experiences suggest otherwise. Study topic, setting, and population might account for some of these differences and therefore merit careful consideration.^48^ The use of online platforms and social media applications (such as the FBMR) for remote data collection has proven helpful in reaching broader audiences,^16 19 21^ but requires close cooperation with ethical review boards to ensure that privacy and confidentiality are maintained.^45 49 50^ Our field experience suggests that remote FGDs, using audiovisual applications, might be a very worthwhile approach but that it can also be an exhausting and intense process that ultimately does not serve to answer a given research question.

### Limitations and opportunities for further research

We are aware of the controversies surrounding the use of FB to collect people’s personal data.^51 52^ The current legal discourse around the ownership of private data on social networks (particularly FB) is complicated^53 54^ and merits discourse that rests beyond the scope of this paper. Our data collection was done via FBMR, with the consent of participants (see informed consent form in online supplemental file 2). We have no knowledge and have found no reliable sources that could outline whether FB records video chats such as those done in FBMR. We ensured, however, that neither the information about the FGD sessions nor information about the participants was posted publicly. The recordings we gathered from the FGD sessions, for the purpose of transcription and data analysis, were recorded using an external application. These video clippings served as data and were therefore accessible to the research team. More practical adaptations could respond to calls for more insight into how we can address overwhelming concerns regarding privacy and confidentiality.

10.1136/fmch-2021-001098.supp2 Supplementary data

## Conclusion

The COVID-19 pandemic forces us to revisit approaches to collecting qualitative data, particularly FGDs.^4 45^ The collection of qualitative data—whether via chat or text-based methods, synchronous or asynchronous, video and/or audio-based approaches, interviews, FGDs or observations—is needed more than ever to ensure that the complexities of family and community health perspectives are seen, heard, documented and translated into programmes and policies.

Shifting from face-to-face to remote FGDs has proven challenging for us, and thus requires enhanced training and test runs to achieve optimal results. FGDs using FBMR are possible, but challenges in participant retention and engagement are considerable and tough to mitigate as participants’ environmental distractions often overpower the flow of the discussion. This is especially true when aiming to engage study populations that often live in close quarters with individuals who are likely to be disruptive (children). Furthermore, we encountered ethical complexities in terms of maintaining participants’ anonymity. In our experiences to date, we are not aware of privacy violations. However, continuous discussions with ethical review boards are critical to ensure that the basic tenets of privacy and confidentiality are maintained.

Despite our efforts to apply a series of adaptations to continue data collection, we ultimately decided that in the current lockdown situation, remote FGDs were not a viable method to address our study objectives. We hope that our experiences will spark a discourse on how to conduct ethical and trustworthy qualitative research during a pandemic, and will promote further development and validation of methodological approaches.

## References

[1] Isaacs A. An overview of qualitative research methodology for public health researchers. Int J Med Public Health 2014;4:318. 10.4103/2230-8598.144055

[2] Scheelbeek PFD, Hamza YA, Schellenberg J, et al. Improving the use of focus group discussions in low income settings. BMC Med Res Methodol 2020;20:287. 10.1186/s12874-020-01168-8 33256625 PMC7706206

[3] Reñosa MDC, Mwamba C, Meghani A, et al. Selfie consents, remote rapport, and Zoom debriefings: collecting qualitative data amid a pandemic in four resource-constrained settings. BMJ Glob Health 2021;6:e004193. 10.1136/bmjgh-2020-004193 PMC7798410 33419929

[4] Hensen B, Mackworth-Young CRS, Simwinga M, et al. Remote data collection for public health research in a COVID-19 era: ethical implications, challenges and opportunities. Health Policy Plan 2021;36:360–8. 10.1093/heapol/czaa158 33881138 PMC7928874

[5] Kitzinger J. Qualitative research. introducing focus groups. BMJ 1995;311:299–302. 10.1136/bmj.311.7000.299 7633241 PMC2550365

[6] Lakshman M, Charles M, Biswas M, et al. Focus group discussions in medical research. Indian J Pediatr 2000;67:358–62. 10.1007/BF02820688 10885209

[7] Kyilleh JM, Tabong PT-N, Konlaan BB. Adolescents' reproductive health knowledge, choices and factors affecting reproductive health choices: a qualitative study in the West Gonja district in northern region, Ghana. BMC Int Health Hum Rights 2018;18:6. 10.1186/s12914-018-0147-5 29361947 PMC5782392

[8] Carrotte ER, Vella AM, Bowring AL, et al. "I am yet to encounter any survey that actually reflects my life": a qualitative study of inclusivity in sexual health research. BMC Med Res Methodol 2016;16:86. 10.1186/s12874-016-0193-4 27465507 PMC4964098

[9] Mkandawire-Valhmu L, Stevens PE. The critical value of focus group discussions in research with women living with HIV in Malawi. Qual Health Res 2010;20:p. 684–96. 10.1177/1049732309354283 19926798

[10] Teshome GS, Modiba LM. Strategies to eliminate mother-to-child transmission of HIV in Addis Ababa, Ethiopia (qualitative study). Hiv Aids 2020;12: :821–37. 10.2147/HIV.S277461 PMC7719320 33293872

[11] Morgan D, Lobe B. Online focus groups, 2011: 199–230.

[12] Tates K, Zwaanswijk M, Otten R, et al. Online focus groups as a tool to collect data in hard-to-include populations: examples from paediatric oncology. BMC Med Res Methodol 2009;9:15. 10.1186/1471-2288-9-15 19257883 PMC2653071

[13] Skelton K, Evans R, LaChenaye J, et al. Utilization of online focus groups to include mothers: a use-case design, reflection, and recommendations. Digit Health 2018;4:205520761877767. 10.1177/2055207618777675 PMC5999054 29942638

[14] Woodyatt CR, Finneran CA, Stephenson R. In-Person versus online focus group discussions: a comparative analysis of data quality. Qual Health Res 2016;26:741–9. 10.1177/1049732316631510 26935719

[15] Reisner SL, Randazzo RK, White Hughto JM, et al. Sensitive health topics with underserved patient populations: methodological considerations for online focus group discussions. Qual Health Res 2018;28:1658–73. 10.1177/1049732317705355 29298574 PMC5758419

[16] Chen J, Neo P. Texting the waters: an assessment of focus groups conducted via the WhatsApp smartphone messaging application. Method Innov 2019;12:205979911988427. 10.1177/2059799119884276

[17] Wirtz AL, Cooney EE, Chaudhry A, et al. Computer-Mediated communication to facilitate synchronous online focus group discussions: feasibility study for qualitative HIV research among transgender women across the United States. J Med Internet Res 2019;21:e12569. 10.2196/12569 30924782 PMC6460306

[18] Wettergren L, Eriksson LE, Nilsson J, et al. Online focus group discussion is a valid and feasible mode when investigating sensitive topics among young persons with a cancer experience. JMIR Res Protoc 2016;5:e86. 10.2196/resprot.5616 27161146 PMC4877501

[19] Biedermann N. The use of Facebook for virtual asynchronous focus groups in qualitative research. Contemp Nurse 2018;54:26–34. 10.1080/10376178.2017.1386072 28975852

[20] Smith TM. Experiences of therapists and occupational therapy students using video Conferencing in conduction of focus groups. Qualitative Report 2014;19:1–13. 10.46743/2160-3715/2014.1233

[21] Colom A. Using WhatsApp for focus group discussions: ecological validity, inclusion and deliberation. Qualitative Research 2021;14:146879412098607. 10.1177/1468794120986074

[22] Dos Santos Marques IC, Theiss LM, Johnson CY, et al. Implementation of virtual focus groups for qualitative data collection in a global pandemic. Am J Surg 2021;221:918–22. 10.1016/j.amjsurg.2020.10.009 33070983 PMC7550163

[23] Reñosa MDC, Wachinger J, Bärnighausen K, et al. How can human-centered design build a story-based video intervention that addresses vaccine hesitancy and bolsters vaccine confidence in the Philippines? A mixedmethod protocol for project SALUBONG. BMJ Open 2021;11:e046814. 10.1136/bmjopen-2020-046814 PMC8190986 34108166

[24] CNN. Metro Manila to be placed on 'lockdown' due to COVID-19, 2020. Available: https://cnnphilippines.com/news/2020/3/12/COVID-19-Metro-Manila-restrictions-Philippines.html?fbclid=IwAR00OkJx4Lee19-BZ9ta_VYUhV6S4sRLqrlVGLFuMh0zKzqcjpnO5KcDKl4

[25] Llorito D. Harnessing digital technologies can help Philippines overcome impact of pandemic, 2020. Hasten Recovery. Available: https://www.worldbank.org/en/news/press-release/2020/10/05/harnessing-digital-technologies-can-help-philippines-overcome-impact-of-pandemic-hasten-recovery

[26] Rayome AD. Messenger Rooms: Here’s how to use Facebook’s free new video chat feature, 2020. Available: https://www.cnet.com/how-to/messenger-rooms-heres-how-to-use-facebooks-free-new-video-chat-feature/

[27] Facebook. Introducing Messenger Rooms and More Ways to Connect When You’re Apart, 2020. Available: https://about.fb.com/news/2020/04/introducing-messenger-rooms/

[28] Egan E. Privacy matters: messenger rooms, 2020. Available: https://about.fb.com/news/2020/04/privacy-matters-messenger-rooms/

[29] O'Flaherty K. Facebook just Launched messenger Rooms—What you should know before using it, 2020. Forbes. Available: https://www.forbes.com/sites/kateoflahertyuk/2020/05/15/facebooks-new-messenger-rooms-launches-why-you-should-think-before-using-it/?sh=56bed98f70d3

[30] Plecher H. Total population of the Philippines 2025, 2020. Available: https://www.statista.com/statistics/578726/total-population-of-philippines/#statisticContainer

[31] Kemp S. Digital 2020: the Philippines, 2020. Available: https://datareportal.com/reports/digital-2020-philippines

[32] Sanchez MJ. Number of Facebook users Philippines 2017-2025, 2020. Available: https://www.statista.com/statistics/490455/number-of-philippines-facebook-users/

[33] Dixon E. People in the Philippines spend the most time online, global report finds, 2019. Available: https://edition.cnn.com/2019/02/01/health/philippines-highest-internet-use-scli-intl/index.html

[34] Abadilla-Dumlao D. At 10 hrs, 2 mins a day, Filipinos spend most time online, in Philippine Daily Inquirer 2019.

[35] Swearingen J. Facebook used the Philippines to test free Internet. then a dictator was elected, 2018. Available: https://nymag.com/intelligencer/2018/09/how-facebooks-free-internet-helped-elect-a-dictator.html

[36] Pagulong CJ, Desiderio L. Facebook offers free Internet access in Phl, 2015. Available: https://www.philstar.com/headlines/2015/03/20/1435536/facebook-offers-free-internet-access-phl

[37] Globe. TM customers can now enjoy free Facebook on their devices, 2015. Available: https://www.speedmagazine.ph/globe-tm-customers-can-now-enjoy-free-facebook-on-their-devices/

[38] David CC, San Pascual MRS, Torres MES. Reliance on Facebook for news and its influence on political engagement. PLoS One 2019;14:e0212263. 10.1371/journal.pone.0212263 30889186 PMC6424427

[39] Martin R. Philippine Journalist Maria Ressa: 'Journalism Is Activism'. NPR 2020.

[40] Wang S. Facebook rules the Internet in the Philippines. Rappler walks the line between partnership and criticism. Nieman Lab, 2017.

[41] Eeuwijk PA. How to … Conduct a Focus Group Discussion (FGD). In: Methodological manual, 2017.

[42] Nyoni P, Velempini M. Privacy and user awareness on Facebook. S Afr J Sci 2018;114. 10.17159/sajs.2018/20170103

[43] Isaak J, Hanna MJ. User data privacy: Facebook, Cambridge Analytica, and privacy protection. Computer 2018;51:56–9. 10.1109/MC.2018.3191268

[44] NerdsChalk Staff. How to join a messenger room, 2020. Available: https://nerdschalk.com/how-to-join-a-messenger-room/

[45] Sy M, O'Leary N, Nagraj S, et al. Doing interprofessional research in the COVID-19 era: a discussion paper. J Interprof Care 2020;34:600–6. 10.1080/13561820.2020.1791808 32718262

[46] Daniels N, Gillen P, Casson K, et al. Steer: factors to consider when designing online focus groups using audiovisual technology in health research. Int J Qual Methods 2019;18:160940691988578. 10.1177/1609406919885786

[47] Tuttas CA. Lessons learned using web conference technology for online focus group interviews. Qual Health Res 2015;25:122–33. 10.1177/1049732314549602 25192765

[48] Brüggen E, Willems P. A critical comparison of Offline focus groups, online focus groups and E-Delphi. International Journal of Market Research 2009;51:1–15. 10.1177/147078530905100301

[49] Ribeiro-Navarrete S, Saura JR, Palacios-Marqués D. Towards a new era of mass data collection: assessing pandemic surveillance technologies to preserve user privacy. Technol Forecast Soc Change 2021;167:120681. 10.1016/j.techfore.2021.120681 33840865 PMC8019834

[50] Saura J, Ribeiro-Soriano D, Palacios-Marqués D. From user-generated data to data-driven innovation: a research agenda to understand user privacy in digital markets. Int J Inform Manage 2021;102331:1–13. 10.1016/j.ijinfomgt.2021.102331

[51] Confessore N. Cambridge Analytica and Facebook: the scandal and the fallout so far, 2018. Available: https://www.nytimes.com/2018/04/04/us/politics/cambridge-analytica-scandal-fallout.html

[52] Lazarus D. Facebook says you ‘own’ all the data you post. Not even close, say privacy experts, 2018. Available: https://www.latimes.com/business/lazarus/la-fi-lazarus-facebook-cambridge-analytica-privacy-20180320-story.html

[53] Schonfeld E. Zuckerberg On Who Owns User Data On Facebook: It’s Complicated, 2009. Available: https://techcrunch.com/2009/02/16/zuckerberg-on-who-owns-user-data-on-facebook-its-complicated-2/

[54] Heller N. We may own our data, but Facebook has a duty to protect it, 2018. Available: https://www.newyorker.com/tech/annals-of-technology/we-may-own-our-data-but-facebook-has-a-duty-to-protect-it

