# Supplementary material for: ‘Unfocused groups’: lessons learnt amid remote focus groups in the Philippines
Source: Fam Med Community Health. 2021 Aug 4;9(Suppl 1):e001098. doi: 10.1136/fmch-2021-001098 (PMC8349646; doi:10.1136/fmch-2021-001098)
Supplement: Supplementary data [file fmch-2021-001098supp001.pdf]

Supplementary Material

Supplementary Table 1. 'FB Messenger Room' and Zoom Built-in Features		
	**FB Messenger Room**	**Zoom**
**Built-in Features**		
**Video**	Participant is in full control of video	Participant is in full control; host can turn it off
**Speaker/Microphone**	Host can mute all participants, only participants can unmute themselves	Host can mute participants, only participants can unmute themselves
**Data Usage**	Approx. 300 mb per hour †	Approx. 810 mb per hour ††
**Subscription and Fees**	Free	Free for users, but paid plan required for FGD host in case the duration should exceed 40 minutes
**Privacy and Security**		
**Logging-in**	Anyone given the link to the room (with and without FB Messenger account) ** can join the room	Anyone given the link/Meeting ID and Password (with and without Zoom account) can join the room
**Anonymity of participants**	No option to change username as participants use their FB/FB Messenger accounts to log in. Users without an account (or who wish to remain anonymous) can 'log-in as guest' and can choose a new username for the discussion<br>Everyone can see the list of participants in the meeting	Participants can change their Caller ID prior to logging-in. Host can rename participants and can disable or enable the participants from changing their Caller ID<br>Everyone can see the list of participants in the meeting
**Removing Participants**	The host can remove (uninvited) participants, even after the meeting has started<br>The meeting can be locked to prevent new participants (invited or not) from entering<br>Participants can invite other people to join	The host can remove (uninvited) participants, even after the meeting has started<br>The meeting can be locked to prevent new participants (invited or not) from entering<br>Participants can invite other people to join
**Meeting Capacity**	Maximum of 50 participants	Maximum of 100 participants
**Time Limit**	No time limit	Time limit of 40 minutes for group sessions, no time limit with paid plans†*
**Scheduling**	Meetings can be scheduled ahead of time	Meetings can be scheduled ahead of time
**Compatibility**	Android, PC, Apple, Mac<br>Can be accessed via internet browser, FB or Messenger software, or mobile application<br>Internet speed should be a minimum of 500kbps, and 1.5mbps for an HD quality video †*†	Android, PC, Apple, Mac<br>Can be accessed via internet browser (mobile web browsers are not supported) [40], or Zoom software/mobile application<br>Internet speed should be a minimum of 500kbps, and 1.5mbps for an HD quality video †*†
**In-Meeting Chats*** **		
**Group Messaging**	The host can only disable in-meeting chats when in mobile mode	The host can disable in-meeting chats
**Private Messaging**	No private messaging available	Private messaging is available, but can be disabled by the host
**Recording**	No recording option available	Recording option available. The host can record the session and enable the participants to record
**Screen Sharing**	Screen sharing is available both in Mobile and in PC mode	Screen sharing is available both in Mobile and in PC mode

†We confirmed the data usage consumption based on our conducted FGDs. Additionally, we refer to: https://www.apps2data.com/insights/how-much-mobile-data-does-video-calling-use/
††https://www.reviews.org/internet-service/how-much-data-does-zoom-use/
**Opening the link via a private browser works and allows the user to choose a different username or alias once entering the room, thereby bolstering privacy and confidentiality.
†*Limited time for Zoom free subscription. Unlimited meeting duration for paid plans.
†*†https://support.zoom.us/hc/en-us/articles/201362023-System-requirements-for-Windows-macOS-and-Linux#:~:text=Bandwidth%20requirements,The%20bandwidth%20used&text=For%201%3A1%20video%20calling,1.8Mbps%20(up%2Fdown
https://www.bandwidthplace.com/internet-speed-facebook-chat-whats-ideal-article/#:~:text=A%20slow%20Internet%20connection%20can,a%20variant%20of%20Skype%20traffic
***In-meeting group chat is available in previous versions of the FB Messenger.
