# Supplementary material for: ‘Unfocused groups’: lessons learnt amid remote focus groups in the Philippines
Source: Fam Med Community Health. 2021 Aug 4;9(Suppl 1):e001098. doi: 10.1136/fmch-2021-001098 (PMC8349646; doi:10.1136/fmch-2021-001098)
Supplement: Supplementary data [file fmch-2021-001098supp002.pdf]

Project SALUBONG
Research Institute for Tropical Medicine, Philippines & University of Heidelberg, Germany

FOCUS GROUP DISCUSSION INFORMATION SHEET

Title of the study:
***SALUBONG*: Building Vaccine Confidence via Empathy and Narrative in the Philippines**

Names of Investigators:
Philippines Principal Investigator:
[redacted]

Germany Principal Investigator:
[redacted]

Name of Sponsor: The Bill and Melinda Gates Foundation

Dear study participant,

We are asking for your participation in a study which is designed to explore perceptions of the public health system in relation to vaccines, the perceptions of vaccines in general, and to develop new ways of communicating information related to vaccination. *This research has been reviewed by the Heidelberg University Ethics Committee and the Research Institute for Tropical Medicine - Institutional Review Board (RITM-IRB).* This study is supported by the Bill and Melinda Gates Grand Challenges foundation. **The following text provides you with information on the background and procedure of the study.** Please read this form carefully. If you have any questions, please ask us. If you decide to participate in this research you will be asked to sign a consent form. A copy of the signed form will be provided to you for your record.

The purpose of this project is to develop a better understanding of parents and community health workers' perceptions, attitudes and experiences of childhood vaccinations. The information you provide may help us to develop ways of communicating information to parents who are making choices regarding the vaccination of their children.

Study Procedures

A focus group discussion will be conducted. It is a kind of group discussion that gathers together people from similar backgrounds to discuss a particular topic. Participants for the said activity are parents/caretakers or minors aged 15-17 with children under-five or Barangay Health Workers. You were purposively selected to be part of this activity. Should you agree to participate, you will be invited in a discussion with other residents of your community with the qualifications mentioned above. Each focus group will be conducted by a trained and experienced focus group moderator and may be done via face-to-face or online. We will audio- and video-record the focus group, and an investigator will also take notes. Each focus group (containing 5-8 participants) will last about 60 minutes. Once the recorded data has been transcribed, it will be anonymized, i.e., none of the recorded data can be linked to any of the participants in the focus groups. After the focus group discussion has been written down, the recording will be destroyed. The data will be analyzed by the coordinating institution of the study, under the leadership of [redacted].

Potential risks

There is little risk in participating in this interview. Some participants can feel that the following may be unpleasant or a burden, because the focus group takes too long or the discussion seems unpleasant, you can stop your participation at any time.

Aligato MF, *et al. Fam Med Com Health* 2021; 9:e001098. doi: 10.1136/fmch-2021-001098

Potential benefits

There are no direct benefits to you. However, this information will be used to guide the way we communicate information about vaccinations to parents in the Philippines. Study results will be presented to stakeholders from the different levels of health care system sometime in early 2021.

Data protection

By signing the informed consent form, you declare that you agree that the principal investigator and his team members collect and process data that is generated in the focus group that you will participate in. We will be assigning you with identification number to keep your participation confidential. The audio- and video-recorded data will be stored on a secure server and will be password protected. All data collected during the focus group, including the audio- and video-recorded data, will be destroyed within five years of collection. The audio- and video-recordings will be deleted as soon as they have been transcribed and the transcripts will contain no information that will allow for the identification of the individual participants. Only members of the research team will have access to the data. We may publish the results of this research, but we will keep any identifying information confidential. This study will comply with the implementing rules and regulations of Philippines RA No. 10173, known as the Data Privacy Act of 2012, to ensure protection of data generated in the research study from any unlawful intervention.

Honorarium and costs

There will be no costs for participation in the study nor will you will be paid. However, we will reimburse the cost of your transportation in attending the focus group discussion (FGD). Likewise, we will be giving refreshments or snacks after the discussion. If done online, we will reimburse the cost incurred from using your mobile data via load cards.

Voluntariness

Participation in this study is voluntary. You have the right – without giving reasons – not to participate in this study, to cancel your given consent, and to terminate your study participation at any time. You have the right to decline to participate before, during or after engaging in this study. Likewise, you can cancel your agreement to process the data collected for this study. Should you decide not to join in this activity, it will not affect your standing in the community nor your rights to any services from the health centre. In case you have further questions, please contact the principal investigators at any time.

Contact for questions

Please feel free to ask about anything you do not understand. Take as much time as you need before you make a decision. If you have any questions about the research, you can ask the research staff authorized by the Philippines Research Institute for Tropical Medicine and University of Heidelberg:

[redacted]

This research has been reviewed and approved by Heidelberg University Ethics Committee and RITM Institutional Review Board, which are the committees whose task is to make sure that research participants are protected from harm**.** If you have questions about your rights as research participants, please feel free to contact:

(details removed in compliance to data privacy)

HEIDELBERG UNIVERSITY HOSPITAL

Project SALUBONG
Research Institute for Tropical Medicine, Philippines & University of Heidelberg, Germany

FOCUS GROUP DISCUSSIONS: INFORMED CONSENT FORM

With my signature, I agree to voluntarily participate in this study. I confirm that I have received the participant information sheet. I received explanations, was able to ask questions and my questions were answered satisfactorily. With my signature, I agree that the focus group discussions that I will participate in will be recorded.

I am giving my voluntary consent to participate in this research.

______________________	______________________
Name of Participant	Signature or thumb print of Participant
______________________	______________________
Date of Signing	Place of Signing

WITNESS (IN CASE THE PARTICIPANT CANNOT READ)

I attest that I have seen and heard the reading and discussion of this document to the participant named above. I also confirm that she was given the opportunity to ask questions and were answered to her satisfaction. I also attest that she/he has provided her/his verbal consent to participate in the study.

______________________	______________________
Name of Witness	Signature of Witness
______________________	______________________
Date of Signing	Place of Signing

PERSON OBTAINING CONSENT

I confirm that I have read this document to the participant and discussed its contents with her/him. I have given her/him the opportunity to ask questions and answered the questions to the best of my ability. I also confirm that s/he gave her/his verbal consent to join in the study.

______________________	______________________
Name of Person Obtaining Consent	Signature of Person Obtaining Consent
______________________	______________________
Date of Signing	Place of Signing

Aligato MF, *et al. Fam Med Com Health* 2021; 9:e001098. doi: 10.1136/fmch-2021-001098
